# Understanding patient transfers across multiple clinics in Zambia among HIV infected adults

**DOI:** 10.1371/journal.pone.0241477

**Published:** 2020-11-04

**Authors:** Kombatende Sikombe, Aaloke Mody, Jillian Kadota, Jesse “Jake” Pry, Sandra Simbeza, Ingrid Eshun-Wilson, Sitali Richard Situmbeko, Chama Bukankala, Laura Beres, Njekwa Mukamba, Mwanza Wa Mwanza, Carolyn Bolton- Moore, Charles B. Holmes, Elvin H. Geng, Izukanji Sikazwe

**Affiliations:** 1 Research Department, Centre for Infectious Disease Research in Zambia, Lusaka, Zambia; 2 Department of Public Health, Environments and Society, London School of Hygiene and Tropical Medicine, London, United Kingdom; 3 Division of Infectious Diseases, Washington University School of Medicine, Washington University in St. Louis, St. Louis, Missouri, United States of America; 4 Division of Pulmonary and Critical Care Medicine and Center for Tuberculosis, University of California San Francisco, San Francisco, California, United States of America; 5 Division of Infectious Diseases, Johns Hopkins University School of Medicine, Baltimore, Maryland, United States of America; 6 Division of Infectious Diseases, University of Alabama, Birmingham, Alabama, United States of America; 7 Center for Global Health and Quality, Georgetown University, Washington, District of Columbia, United States of America; University of the Witwatersrand, SOUTH AFRICA

## Abstract

Many patients in HIV care in Africa considered lost to follow up (LTFU) at one facility are reportedly accessing care in another. The success of these unofficial transfers as measured by time to re-entry at the new-facility, prevalence of treatment interruptions, speed of ART-initiation, and overall continuity of care is not well characterized but may reveal opportunities for improvement. We traced a random sample of LTFU HIV-infected patients in Zambia. Among those found alive and reported in care at a new-facility, we reviewed records at the receiving facility to verify transfer; and when verified, documented the transfer experience. We used Kaplan-Meier methods to examine incidence of ART-initiation after transfer to new clinic. We assessed demographic and clinical characteristics, official and cross-provincial transfer for associations with HIV treatment re-engagement using Poisson regression models and associations between official-transfer and same-day ART initiation at the new-facility. Among 350 LTFU-patients, 178 (51%) were successfully verified through chart review at the new-facility. 132 (74.2%) were female, 72 (40.4%) aged 25–35, and 51% were ever recorded as previously being on ART. 110 patients (61.8%) were registered under new ART-IDs and 97 (54.5%) received a new HIV test. 54% of those previously on ART-initiated on the same-day. Using the same ART-ID was associated with same-day initiation compared to those receiving a new ART-ID (p = 0.07). 80% (n = 91) of those ever on ART had evidence of medication initiation at new clinic. Among these, initiation reached 66% (95% CI: 56–75) by 30 days, 77.5% (95% CI: 68–86) by 90 days after new-facility presentation. Many patients use new identifiers at new facilities, indicative of inefficiencies. Re-entry into new facilities among the unofficial-transfer population is often delayed and timely treatment initiation is inconsistent, suggesting interruptions in treatment. Health systems innovations to ensure smooth and safe transfers are needed to maintain quality HIV care.

## Introduction

In the current era of universal treatment for all persons infected with HIV, poor retention in care remains a substantial barrier to optimizing viral suppression [1]. Although routine programmatic data often indicate high level of lost to follow-up (LTFU)- particularly where systems are paper-based with no unique patient identifiers (IDs), several prior studies have demonstrated that many patients considered LTFU at their original facility are in fact accessing care in another [2–9]. Though encouraging, current data only speak to whether a patient has eventually transferred to a new facility or not. Unfortunately, current systems are poorly equipped to accommodate the smooth transfer and tracking of patients from one facility to the next due to the lack of unique patient IDs, multiple implementing partners and unified health system records [10]. Thus, transfers may not be seamless, but it is not known to what extent they undermine the significant clinical benefits of early and uninterrupted HIV treatment.

As HIV care is life-long and mobility is a normal part of livelihood and social practices, improving our understanding of the experience and safety of transfers across facilities is imperative for informing how to optimize receipt of patients previously on treatment elsewhere [11]. Existing data suggests that the time between the last visit at the original facility and the next visit at the receiving facility is often delayed: one recent study describing the patient transfer experience among people living with HIV (PLHIV) in Kenya showed that treatment gaps greater than 14 days were common [4]. This study also showed that rate to reengagement differs by whether transfers are managed at the clinic level or ad-hoc by the patient (i.e., documented, official versus undocumented, unofficial transfers); for example, patients who left with an official transfer letter reengaged in care six times faster than patients who did not. Further evidence indicates that time to ART-initiation may vary by the distance from home to the clinic where care is re-accessed [8]. These findings are suggestive of gaps in care associated with patient transfer and differential rates of treatment initiation according to where patients transfer and how and/or if they were managed appropriately. Further evidence on what happens to patients after they transfer, such as understanding the engagement process e.g. repeat HIV testing, use of unique identifiers and CD4 testing is not well known.

To address these gaps, we undertook an assessment of patients who were LTFU according to EMR records at their original facility and reported having transferred to a new facility after being traced in the community in Zambia. We examined medical records at the receiving facility to verify patient-reported transfer and documented the transfer process including whether patients registered under new names or ART IDs, whether the receiving facility appropriately and rapidly staged and treated patients, and distances between original and new facility. We characterized the incidence of ART-initiation between facilities and assessed whether the rate of initiation was associated with transferring officially and/or across a province. Finally, we evaluated characteristics associated with same-day ART-initiation at the transfer/new clinic.

## Materials and methods

### Patient population

The target population for this study are adults (≥18 years) living with HIV infection in Zambia who had transferred to a new facility. Our study population consisted of patients who were considered LTFU from HIV care (i.e., 90 days late to an appointment at the time of sampling) at their original clinic based on EMR records, reported transferring to a new facility after being actively traced in the field, and whose transfer was then verified at the new facility as part of study activities for the *Better Information for Health in Zambia* cohort study [1]. The study undertook a multistage sampling campaign and active tracing of a random sample of patients LTFU in order to obtain regionally representative estimates of retention and mortality. We selected using probabilities proportional to size a minimum of 2 to 10 facilities from each of 12 strata defined by facility type and province for a total of 32 government-operated HIV treatment clinics in four provinces (Lusaka, Southern, Eastern, Western) that received technical assistance support from the Centre for Infectious Disease Research in Zambia (CIDRZ), a Zambian non-governmental organization. In each selected facility, we enumerated all adults on ART who made a visit in the previous 24 months and identified lost to follow-up from this. From patients lost to follow-up, we selected a simple random sample for tracing inverse proportional to size.

### Procedures and measurements

We hypothesise that some patients considered LTFU in one clinic are in fact seeking care elsewhere. Among all patients who made at least one clinic visit between August 1, 2013 and July 31, 2015 to one of the study facilities, we actively traced in the community a random sample of patients considered LTFU as of July 31, 2015 and administered a LTFU questionnaire that included questions regarding whether they had transferred to a new facility, to which facility they had transferred, the date of their first visit to the new facility, and their reasons for transfer. Of note, patients who had a transfer to another facility with official documentation that was documented in the EMR were not considered LTFU and were not included in our population. We defined patients as “Previously on ART” if they had ever previously been initiated on ART at their original clinic as per national ART guidelines. “Not on ART” or “Never yet initiated on ART” was used to define those who had tested positive for HIV at their original clinic but had never initiated ART based on the CD4 count threshold as per national ART guidelines at the time [12]. Per our usage, someone who previously initiated on ART but then had a treatment interruption was still considered as “Previously on ART”. Among those patients who did report transferring their care to a new facility, tracers attempted to then verify the transfer by going to the receiving facility and abstracting the patient health records through a combination of EMR and paper chart review. Patients were matched using a combination of treatment identifier numbers, name, and dates of birth at the receiving facility. Data collection included information on whether there was official transfer documentation from the original clinics, whether patient treatment identifiers were preserved (i.e. same ART ID used at the prior site also used at receiving site), and the date of ART-initiation at the new clinic for those who had previously initiated ART. We defined ART-initiation as a patient starting ART at a new receiving clinic regardless of them not having any treatment interruptions during transfer. Verification was not attempted in facilities in provinces that were not supported by CIDRZ as study sensitization was not possible nor in clinics under the Ministry of Defence. All other sociodemographic (sex, age), clinical (initial date of ART initiation, scheduled/ attended visit dates, CD4 counts), and facility-level data (GPS coordinates) were obtained from the EMR.

### Statistical analysis

We summarized patient and transfer characteristics using counts and proportions for categorical variables, and medians and interquartile ranges (IQRs) for continuous data. To estimate the transfer distances, we geocoded clinic locations using latitude and longitude measure, mapped transfers using QGIS 2.1.8 with standardized world geodetic system 1984 (Free Software Foundation, Inc. Boston, MA 02110–1301 USA) using GADM (gadm.org University of California, Davis U.S.A.) administrative boundary layers v2.8 (accessed November 2019), and used the Vincenty formula to measure the distance in kilometres between clinic locations [13]. The map used is not copyrighted and allowed to be used freely.

To characterize time delays in transfers, we describe the percent of patients who had successfully transferred over time since the date of their last scheduled pharmacy pickup date/ last scheduled visit at the previous/original facility, which was used to approximate the last date with which the patient was in possession of ART. For patients who were missing a scheduled pharmacy pickup (n = 21), we used the mean time to next pharmacy pickup at their original clinic to impute their next scheduled pickup. We fit a modified Poisson regression with robust variances to assess the relationship between having no treatment interruption, defined as transferring to the new facility prior to being 14 days late to their previously scheduled pharmacy pickup, with sociodemographic, clinical, and transfer characteristics. Covariates included in the multivariable model were selected based on *a priori* hypotheses driven by previously identified causal relationships using directed acyclic graphs to identify confounders (S1 Fig) [2, 4, 7, 14].

To assess re-engagement of patients after transferring to the new facility, we estimated the cumulative incidence of ART-initiation at the new clinic using the Kaplan-Meier method among patients who had previously been initiated on ART. Time zero was the date of the first visit at the new clinic and patients were censored at the date of ART start (based on pharmacy records) or administratively censored at the time of record review at the new facility (i.e., May 2016). We then fit a modified Poisson regression to assess the relationship between being initiated on ART at the first visit after transfer with sociodemographic and transfer characteristics based on *a priori* hypotheses of causal relationships.

### Ethics

Ethical approval was obtained from the University of Zambia Biomedical Research Ethics Committee (UNZABREC), University of Alabama at Birmingham (UAB) institutional review boards (IRB) and Ministry of Health (MOH) prior to initiating study procedures. All participants provided written informed consent which was recorded on the consent form. All IRBs approved this consent procedure.

## Results

**Fig 1:**
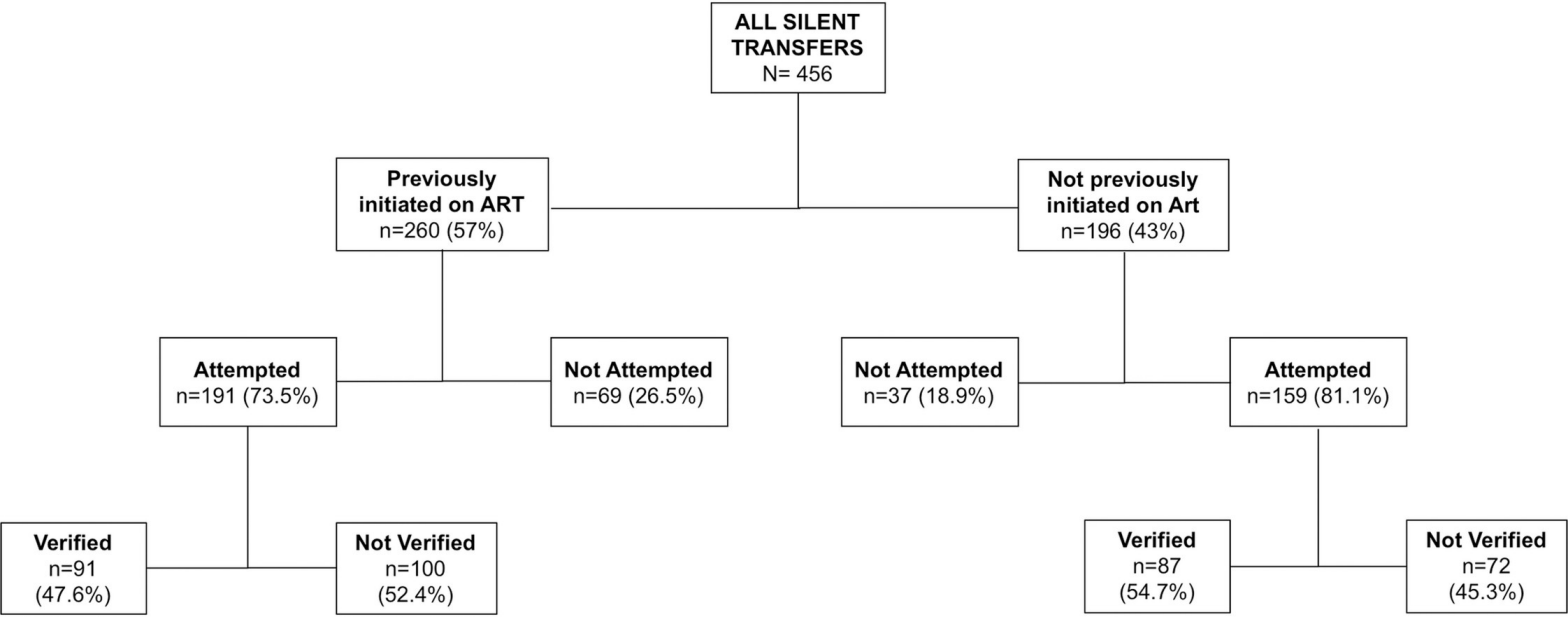
Study population flowchart. Transfers undocumented at original facility for patients previously on ART and those previously not initiated on ART (N = 456).

As described in the parent study, at the 32 selected sites, 104,966 patients made any visit between August 1, 2013 and July 31, 2015 and 17,602 (17%) were LTFU [1]. We selected a random sample of 2,892 lost patients (16% of 17,602 lost patients at 32 selected facilities) for intensive tracing to ascertain current care status. Among the 2892 patients who were traced in the field, updated information was found for 2,163 (75%), of whom 1,751 (81%) were alive. Among those found alive, 456 (26%) had transferred to another facility but were originally captured as LTFU in EMR at original facility (Fig 1). Among the 456 LTFU patients who were traced in the field and reported transferring to a new facility 260 (57.0%) had previously initiated ART, (65.1% female, median age 34 years [IQR 29–40], median CD4 272 cell/μL [IQR 139–445]), we attempted to verify 350 (77%) of these transfers and successfully verified 178 out of 350 (51%) (Table 1). Of these 178, 91 (51.1%) had initiated ART at their original facility and the rest 87 (48.9%) were pre-ARTs and had not yet initiated ART at their original facility. We did not attempt to verify reported transfers in 106 out of 456 (23%) patients due to the patient transferring to a clinic that was managed by different partners and not affiliated with CIDRZ and to which we do not have access to medical records. Among the patients in whom we attempted to verify their transfer, we were slightly more likely to verify transfers among female patients, patients from rural clinics, and patients with a lower-middle basic education level. In contrast, we were less likely to verify unofficial transfers in patients with lower CD4s, urban populations, and patients with upper-basic/secondary education (Table 1).

**Table 1:** Patient characteristics of all transfers identified in person.

Patient Characteristics		All Transfers (n = 456)	Attempted to verify (n = 350)	Verified transfers (n = 178)	Not Verified (n = 172)	p-value
**Sex**	Female	297 (65.1)	242 (69.1)	132 (74.2)	110 (64.0%)	0.039
**Age category**	<25	69 (15.1)	60 (17.1)	29 (16.3)	31 (18.0%)	0.91
	25-35y	179 (39.3)	137 (39.1)	72 (40.4)	65 (37.8%)	
	35-50y	169 (37.1)	125 (35.7)	64 (36.0)	61 (35.5%)	
	>50y	39 (8.6)	28 (8.0)	13 (7.3)	15 (8.7%)	
**Enrollment CD4 cell count category cell/μL**	<200	115 (25.2)	82 (23.4)	34 (19.1)	48 (27.9%)	0.36
	200–350	88 (19.3)	58 (16.6)	29 (16.3)	29 (16.9%)	
	350–500	55 (12.1)	45 (12.9)	26 (14.6)	19 (11.0%)	
	>500	69 (15.1)	53 (15.1)	25 (14.0)	28 (16.3%)	
	Missing	129 (28.3)	112 (32.0)	64 (36.0)	48 (27.9%)	
**WHO Stage at care initiation**	1	203 (44.5)	162 (46.3)	84 (47.2)	78 (45.3%)	0.60
	2	80 (17.5)	66 (18.9)	38 (21.3)	28 (16.3%)	
	3	98 (21.5)	66 (18.9)	33 (18.5)	33 (19.2%)	
	4	18 (3.9)	13 (3.7)	5 (2.8)	8 (4.7%)	
	Missing	57 (12.5)	43 (12.3)	18 (10.1)	25 (14.5%)	
**Marital status**	Single	71 (15.6)	50 (14.3)	23 (12.9)	27 (15.7%)	0.26
	Married	272 (59.6)	214 (61.1)	105 (59.0)	109 (63.4%)	
	Divorced	64 (14.0)	48 (13.7)	29 (16.3)	19 (11.0%)	
	Widowed	42 (9.2)	32 (9.1)	20 (11.2)	12 (7.0%)	
	Unknown	7 (1.5)	6 (1.7)	1 (0.6)	1 (0.6%)	
**Education level**	None	22 (4.8)	22 (6.3)	7 (3.9)	15 (8.7%)	<0.001
	Lower-mid basic	166 (36.4)	141 (40.3)	92 (51.7)	49 (28.5%)	
	Upper basic/Secondary	207 (45.4)	154 (44.0)	65 (36.5)	89 (51.7%)	
	College/University	52 (11.4)	24 (6.9)	10 (5.6)	14 (8.1%)	
	Missing	9 (2.0)	9 (2.6)	4 (2.2)	5 (2.9%)	
**Disclosure status**	Yes	408 (89.5)	309 (88.3)	158 (88.8)	151 (87.8%)	0.45
**Facility type**	Rural	104 (22.8)	90 (25.7)	59 (33.1)	31 (18.0%)	0.003
	Urban	230 (50.4)	165 (47.1)	71 (39.9)	94 (54.7%)	
	Hospital	122 (26.8)	95 (27.1)	48 (27.0)	47 (27.3%)	
**Province**	Eastern	69 (15.1)	66 (18.9)	37 (20.8)	29 (16.9%)	0.006
	Lusaka	168 (36.8)	99 (28.3)	41 (23.0)	58 (33.7%)	
	Southern	137 (30.0)	113 (32.3)	52 (29.2)	61 (35.5%)	
	Western	82 (18.0)	72 (20.6)	48 (27.0)	24 (14.0%)	
**Previously on ART**	Yes	260 (57.0)	191 (54.6)	91 (51.1)	100 (58.1%)	0.19
**Median time to LTFU (IQR)**		0.7 (0.0, 2.8)	0.7(0.0,2.7)	0.7 (0.0, 2.7)	0.7 (0.0, 2.7)	0.63

Values N (%) or Median (IQR), WHO; World Health Organisation, ART; Antiretroviral Therapy, LTFU; Lost to follow up.

### Transfer characteristics

**Fig 2:**
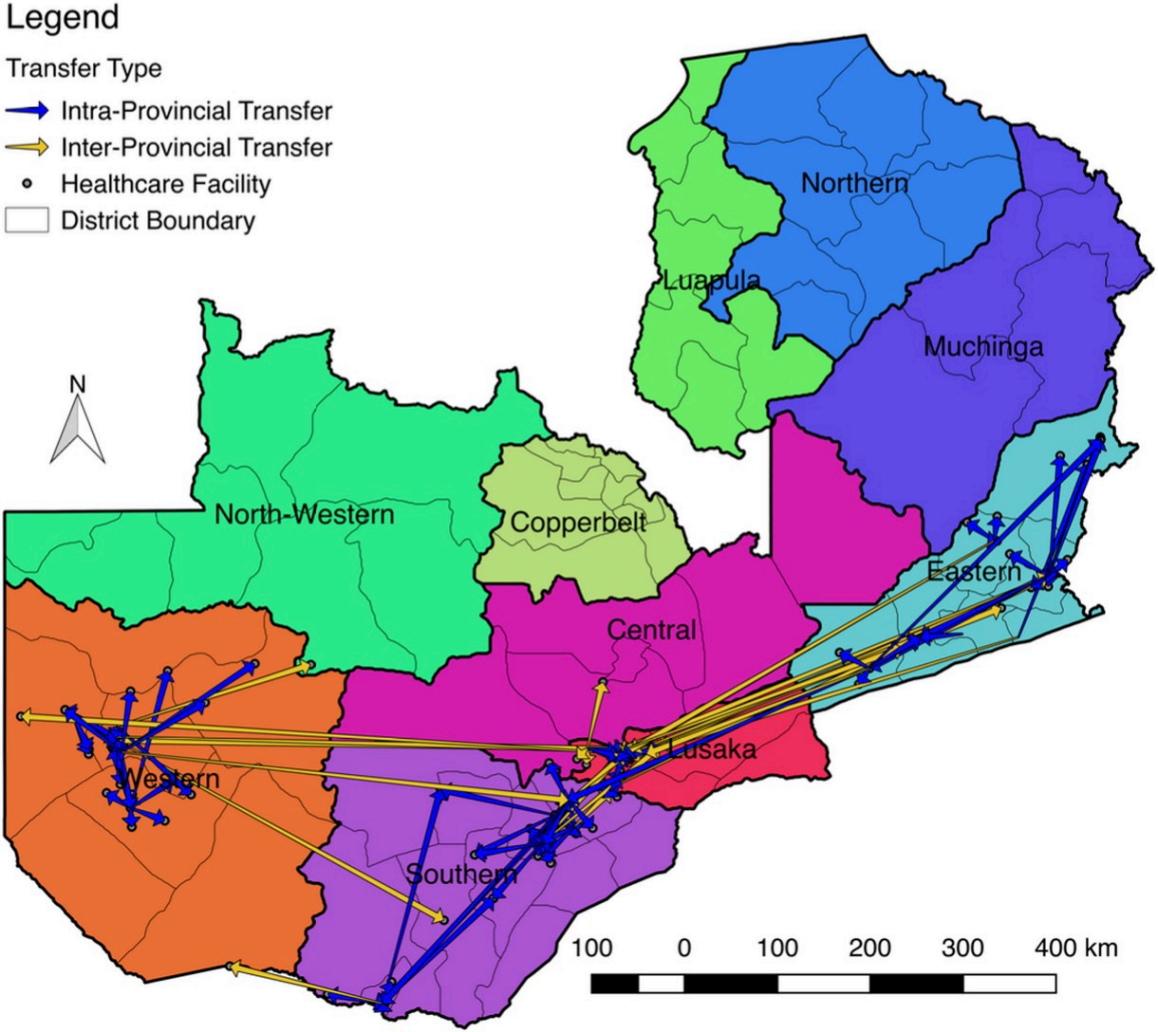
Transfers across study provinces. Inter and intra provincial transfer of patients originally identified as silent transfers.

Among the total verified transfer population, the median gap between last scheduled pharmacy pick up visit/ last scheduled clinic visit at the original facility and first clinic visit at the new facility was 188 days (IQR: 44–446) (Table 2). 46 out of 178 (25.8%) had official transfer paperwork from the original facility (i.e., official transfer documentation in new clinic paper record but not captured in EMR at original facility). At the receiving facility, 68 (38.2%) patients used the same ART ID, 97 (54.5%) received a new HIV test, and 57 (32.0%) received a new CD4 test within 3 months of transferring. 49 (54.0%) of all 91 patients who had previously initiated ART were initiated on ART the first day at the new facility (compared to 35 (40%) out of 87 patients who had not previously been on ART). Only 29 (65.9%) out of 44 patients who had been on ART and had official transfer documentation were initiated on the same day. A greater proportion of patients previously on ART were documented as official transfers (43%, n = 39 out of 91) and used their previous ART number (n = 59, 65%) as compared to those not previously on ART (n = 7, 8% and n = 9, 10%, out of 87 respectively) Table 2. Fig 2 shows transfers based on geocoded clinic locations. 38 (21.3%) of all 178 verified transfers transferred cross-provincially; the median distance between clinics was 27.1km (IQR 7.6, 76.0).

**Table 2:** Transfer characteristics of those identified at receiving facility.

	Study Population						
	All verified transfers n = 178	ART Status			Transfer Status		
		Previously initiated on ART n = 91	Never yet initiated on ART n = 87	p-value	Official transfer n = 46	Unofficial Transfer n = 131	p-value
**Province of origin clinic**							
**Lusaka**	41 (23.0)	19 (21)	22 (25)	0.87	7 (15.2)	34 (26.0)	0.31
**Eastern**	37 (20.8)	20 (22)	17 (20)		12 (26.1)	24 (18.3)	
**Western**	48 (27)	26 (29)	26 (30)		11 (23.9)	36 (27.5)	
**Southern**	52 (29.2)	26 (29)	22 (25)		16 (34.8)	37 (28.2)	
**Cross Provincial Transfer**	38 (21.3)	23 (25%)	15 (17)	0.19	14 (30.4)	24 (18.3)	0.085
**Median time to transfer (days), (IQR)**	188.0 (44.0,446.0)	155.0 (21.0,436.0)	270.0 (115.7,449.0)	0.016	119.5 (-2.0, 434.0)	232.0 (92.0,446.0)	0.034
**Median transfer distance between clinics km, (IQR)**	27.1 (7.6, 76.0)	47.6 (17.8,151.0)	21.8 (3.0, 45.5)	<0.001	58.0 (23.1,239.9)	23.3 (5.5, 57.5)	0.003
**Type of Transfer**							
**Hospital to Hospital**	19 (10.7)	14 (15)	5 (6)	0.26	6 (13.0)	13 (9.9)	0.86
**Hospital to Rural**	14 (7.9)	5 (5)	9 (10)		5 (10.9)	9 (6.9)	
**Hospital to Urban**	17 (9.6)	7 (8)	10 (11)		4 (8.7)	13 (9.9)	
**Rural to Hospital**	17 (9.6)	8 (9)	9 (10)		2 (4.3)	15 (11.5)	
**Rural to Rural**	28 (15.7)	14 (15)	14 (16)		6 (13.0)	22 (16.8)	
**Rural to Urban**	8 (4.5)	4 (4)	4 (5)		2 (4.3)	6 (4.6)	
**Urban to Hospital**	17 (9.6)	12 (13)	5 (6)		5 (10.9)	12 (9.2)	
**Urban to Rural**	19 (10.7)	11 (12)	8 (9)		6 (13.0)	13 (9.9)	
**Urban to Urban**	25 (14.0)	10 (11)	15 (17)		5 (10.9)	19 (14.5)	
**Unknown**	14 (7.9)	6 (7)	8 (9)		5 (10.9)	9 (6.9)	
**Prior ART**	91 (51.1)	-	-		39 (84.8)	52 (39.7)	<0.001
**Official transfer**	46 (25.8)	39 (43)	7 (8)	<0.001	-	-	
**Same ART ID new site**	68 (38.2)	59 (65)	9 (10)	<0.001	41 (89.1)	27 (20.6)	<0.001
**Received new HIV test**	97 (54.5)	27 (30)	70 (80)	<0.001	2 (4.3)	95 (72.5)	<0.001
**New CD4 test cell/μL**	57 (32.0)	24 (26)	33 (38)	0.099	11 (23.9)	45 (34.4)	0.19
**Same day ART initiation**	84 (47.2)	49 (54)	35 (40)	0.069	25 (54.3)	58 (44.3)	0.24

All numbers are in n (%) or Median (IQR). Values N (%) or Median (IQR), WHO; World Health Organisation, ART; Antiretroviral Therapy, LTFU; Lost to follow up.

### Time to transfer

**Fig 3:**
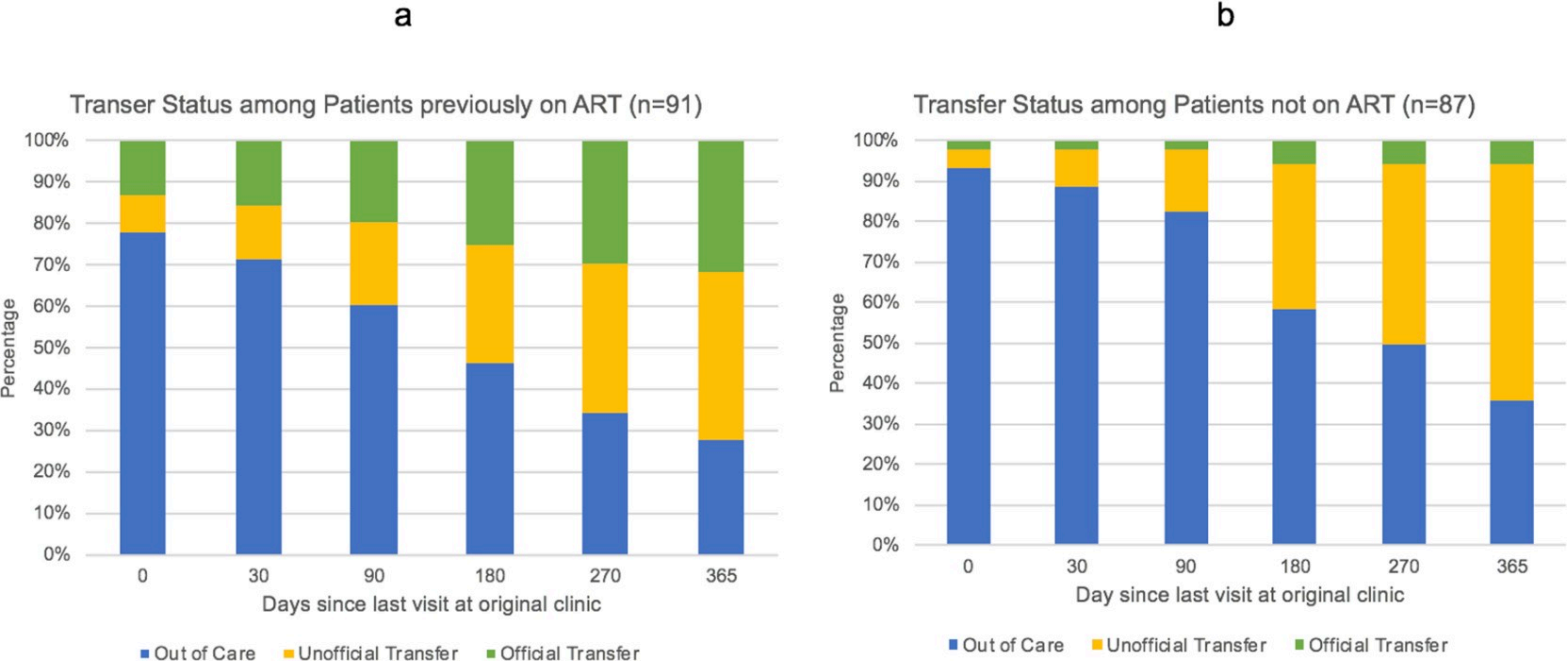
Transfer status among patients. A) Time to transfer from last visit at original clinic among patients previously on ART. B) Time to transfer from last visit at original clinic among patients not previously on ART.

Among patients previously initiated on ART, only 49 (53.8%) and 66 (72.5%) out of 91 had transferred to the new facility by 6 months and 12 months after their last scheduled appointment at their original facility, indicating that a majority of patients experienced a prolonged gap in medications and were out of care, meaning they had not yet presented to the new clinic (e.g. 42 (46.2%) and 25 (27.5%) out of 91 at 6 months and 12 months) despite eventually transferring to a new facility (Fig 3A). For patients never yet initiated on ART, 36 (41.4%) and 56 (64.4%) out of 87 had transferred to the new facility by 6 months and 12 months after their last scheduled appointment at their original facility (Fig 3B). Only 6 (6.9%) of patients never yet initiated on ART and 19 out of 91 (20.9%) of patients previously initiated on ART had transferred without experiencing a gap in their care (i.e., transferred prior to their next scheduled appointment).

In multivariate Poisson regression, we observed those that had official transfer paperwork had a lower risk (RR 0.79, 95% CI 0.63–0.98, p = 0.04) of experiencing a medication gap of ≥14 days (i.e., no gap in care) (Table 3.).

**Table 3:** Results of multivariate Poisson regression evaluating predictors of transferring within 14 days of next scheduled appointment, all verified transfers (n = 178).

Medication gap of 14 days	RR (95% Confidence Interval)	P-value
**Male sex**	1.06 (0.92–1.22)	0.39
**Age at last visit, per year increase**	1.00 (0.99–1.01)	0.37
**Officially transferred**	0.79 (0.63–0.98)	0.04
**Previously on art**	0.87 (0.74–1.03)	0.11
**Time to LTFU**	1.00 (0.96–1.04)	0.93
**Cross provincial transfer**	1.05 (0.90–1.22)	0.48

### Time to ART-initiation among patients previously on ART

**Fig 4:**
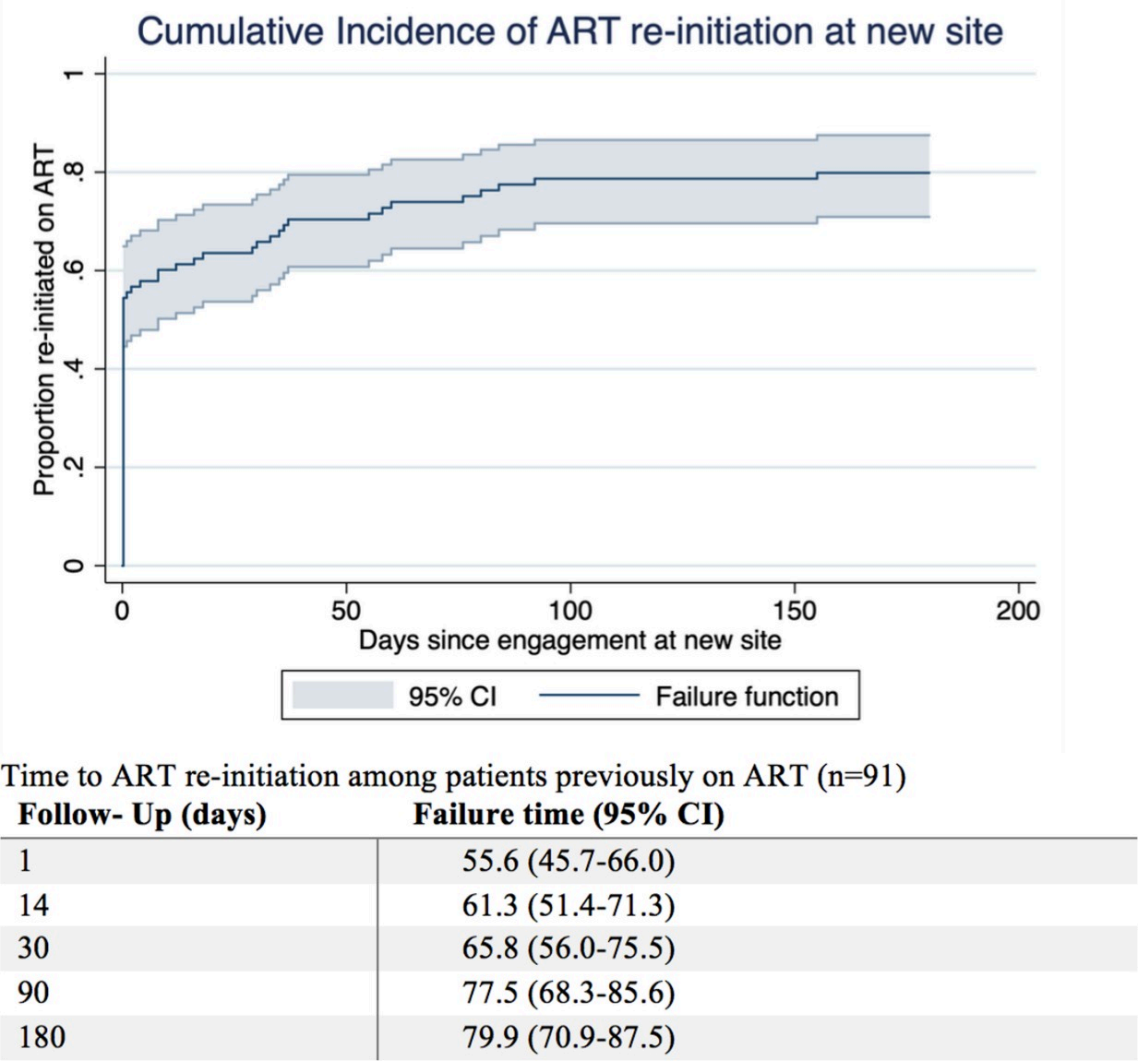
Kaplan-Meier estimates of ART-initiation at new site. Cumulative incidence of ART-initiation by 14, 90, and 180 days since transfer to new site (n = 91).

Based on Kaplan-Meier estimates, 61.3% (95% CI 51.4–71.3%), 77.5% (68.3%-85.6%), and 79.9% (70.9–87.5%) of patients who had previously been on ART had been started on ART at the new clinic by 14, 90, and 180 days after new clinic presentation. Only 55.6% (95% CI 45.7–66.0) were initiated on ART on the day of transfer while 20.1% (95% CI) had not yet been initiated on ART by 180 days after transfer despite previously having been on ART (Fig 4).

In multivariate Poisson regression to assess same-day ART-initiation, using the same ART ID from the previous clinic (RR 1.83, 95% CI 1.8–3.12, p = 0.026) was associated with starting ART on the same day among patients who had previously been on ART (Table 4).

**Table 4:** Multivariate Poisson regression to assess same-day ART-initiation.

Same day initiation	IRR	Std. Err.	z	P>|z|	[95% Conf. Interval]	
Male	0.93	0.18	-0.38	0.71	0.64	1.35
Age at last visit	0.98	0.11	-1.65	0.09	0.96	1.00
Official transfer						
Yes	1.02	0.19	0.11	0.91	0.70	1.49
Used same ART id						
Yes	1.83	0.49	2.23	0.02	1.07	3.11
Cross Provincial transfer	0.92	0.18	-0.39	0.69	0.62	1.38
Medication gap _ 14days	1.67	0.47	1.81	0.07	0.95	2.92
_cons	0.46	0.24	-1.47	0.14	0.16	1.29

## Discussion

We found evidence of engagement in care at the receiving clinic for patients originally classified as LTFU, with over half of transfers we attempted to verify contacted in person and verified as receiving care at an alternate facility. Our findings revealed evidence suggestive of potential poor transfer management at original and receiving sites, substantial gaps between treatment visits, and rates to ART initiation which varied by important transfer characteristics. In this study, patients who transferred from their original clinic were either “official transfers”, in which a letter from original to receiving clinic was provided, or unofficial transfers/ “silent transfers”, whereby no formal transfer documentation process was followed [15]. Using this definition and according to our original clinic assessment using the EMR, we originally identified all 91 patients previously on ART as unofficial transfers, however, upon data abstraction activities at the receiving clinic, we found that approximately 43% of the 91 had in fact transferred with an official letter: indicating misclassification of patient status in the EMR. Misclassifications of patient status indicate issues with data quality at both original and receiving facilities. Because at the time in Zambia, patient records were largely first documented on paper and later entered electronically (i.e. “e-last”), a potential reason for this misclassification could have been the result of delayed transfer of information from the paper record into the electronic record.

Our transfer verification process demonstrated that- while patients transferring officially experienced shorter average gaps in treatment between sites- most patients still experience a prolonged treatment lapse, a similar finding to Hickey et al. [4]. Other important insights from our verification process, which involved abstraction of pharmacy and clinical records at both the previous clinic and the receiving clinic of transfer, revealed a median treatment gap of over half a year for all transfers regardless of ART status. Such significant gaps in treatment due to slow transition between sites are a cause for serious concern, as any relapse in treatment represents vulnerability to virologic rebound [16–18]. Overall, our findings from our verification process illustrate the importance of not only considering patient outcomes once received at a new facility, but rather their entire process of accessing and transitioning care from one facility to the next. By examining this process and relevant patient outcomes, we were able to paint a fuller picture of patient transfers hence strategies that address or expedite this transfer process in a timely fashion, thereby avoiding treatment failure are needed. Thus, while official transfer letters may expedite ART-initiation once patients have successfully re-engaged at a new clinic, this finding indicates that transition of care across facilities remains a challenge [2–4, 7, 8, 19, 20].

Our verification assessment revealed other important administrative and clinical inefficiencies. For example, among the patients we were able to verify at transfer sites, we found that just over half received a new HIV test and just under two thirds received a new ART ID. In the often overcrowded and overburdened HIV care clinics both in Zambia and in SSA at large, time spent with providers is perhaps one the most precious of the limited resources [21]. Particularly in light of the current health care worker shortage crisis in Africa [22, 23], such clinical inefficiencies represent an important area of improvement. Further, as ART services continue to expand with the adoption of universal test and treat [12] the availability of treatment support could even further diminish with a bourgeoning population of patients to serve, making efficiency an even more important priority.

We found evidence for factors associated with ART-initiation even after successful re-engagement with care: among patients who ever initiated, some failed to begin ART even up to 180 days since new clinic presentation. Distance of transfer, especially among those transferring across provinces, could be a potential factor inhibiting swift ART initiation through sub-optimal patient confirmation/identification processes at the facility-level. Nevertheless, it was encouraging to find that in our study population, over 55% of patients who transferred initiated ART on the same day they presented at the receiving clinic. Our mixed-effects Poisson regression model revealed that among patients previously on ART once at the new site, using the same ART I.D from the previous clinic had 1.8 times the odds of same-day initiation compared to those without an ART I.D. Based on our knowledge of patient transfers in Zambia, some of this could be due to the current process where patients sometimes carry a box of their drugs when they report to the new facility or having some form of paperwork therefore making their transfer seamless. The fact that these patients were recorded as LTFU at their original facility shows that smooth management of a formal transfer process is driven by patients. In some facilities, it has been reported that patients sometimes collect drugs under false names hence it’s common for some providers to treat patients as new HIV patients prior to ART start and this is sometimes based on the behaviour/ stigma in their clinic catchment population [24]. Therefore, there are important areas for improvement at the facility level such as better communication with the receiving facility, better provider patient relationships and ensuring complete records are transferred to prevent unnecessary delays in seeking care, but also ensuring that the original facility records the transfer in the EMR.

We were unable to verify just under 50% of transfers perhaps because patients may have registered to their new clinic under a falsified name or acted as a new patient and subsequently registered under a new ART ID, perhaps to avoid being reprimanded by clinic staff, or due to a desire to maintain a level of anonymity [2, 4, 8, 24, 25]. Transitioning to an alternate site similar to their original clinic with regard to clinic type (e.g. rural to rural) suggests a clinic shopping phenomenon whereby patients elect to seek care at other facilities within their community, something which has also been reported by others for within province transfers [2, 9]. To mitigate the challenges of patient mobility, strategies that allow for use of unique patient identifiers would be beneficial to managing ART programs and accurately accounting for retention. Such strategies could include having a standard unique patient identifier at the level of the national health system. Addressing the challenges of patient mobility may require national adoption of mandatory issuance of a national registration numbers at birth [26]. This number can then serve as a unique patient identifier for the entire health system and can be used as a primary key for data linkages between facilities and various government registries and programs [3]. For example, in quantifying the reasons for these verification failures, we found that the majority were due to inability to find the patient by name, ART ID, or a combination of the two in paper and/or electronic records at the receiving clinic. The reasons for these failures could be due to a lack of organization at new sites, whereby paper files were missing and/or not available–a common occurrence in HIV care clinics in Zambia [27]. It could also be that patients did indeed access care at the indicated transfer facility, but poor case management at the receiving site led to the generation of a new patient ART ID and therefore making him/her impossible to identify in our verification process. Improved data platforms that ensure better management of electronic records would reduce the demand on the health system in restaging and re-testing for blood–a suggestion also reported by Hickey et al [4]. Integrating patient records into unique identifiers will improve the quality and continuity of care without having to repeat staging or ART initiation procedures–time and resource-saving practices which are especially crucial given the healthcare worker shortage crisis in SSA. Importantly, the gradual transition to “e-first” systems, whereby patient information is first entered electronically, may be an important first step towards alleviating similar issues related to lack of information sharing across facilities and/or delayed data entry in the future.

With the adoption of universal test and treat [12], it is possible that treatment support could even further diminish over time with increasing clinic size. Patient services may focus more on treatment initiation with less focus on LTFU and patients that transfer [14, 28]. However, our findings instead demonstrate the importance of this “silent transfer” group to the ultimate success of ‘90-90-90’: it is imperative to continue to describe the entire patient transfer experience across clinics, understand the challenges they face, and explore options for improvement. To our knowledge, only one other study has reported on the time to reengagement in care following a silent transfer [4], and the other on the time to reengagement following official transfers in low resource settings [11]. Ours is the first study that assessed transfer characteristics cross-provincially and within province. Other study strengths include our enhanced and intensive patient tracing approach, which encompassed verification of transfers at the indicated transfer facility across four provinces in Zambia. We maintain that, while demanding, undertaking this approach led to a better overall understanding of the patient transfer experience. Future research implementing this method in other contexts may reveal additional opportunities for improvement.

## Conclusions

Given that we found that patients originally considered LTFU in the original facility had transferred to a new facility and were accessing care, platforms that are able to uniquely identify patients, allowing for better coordination and easy transition of patients from one facility to another, as well as policies/procedures that are more flexible to patient clinic transfer are lacking and/or require much improvement in the current context of HIV care in Zambia. Further research that explores whether patients we were unable to verify at a new facility may have indeed returned to care at original facility is required.

We speculate that improved facilitation of patient transfers will be imperative for preserving the positive impact of ART for PLHIV and for the larger population as a whole. A nationally comprehensive database and unique identifiers linking patient records over multiple years can provide a more complete dataset than those available to researchers and planners. For example, use of unique identifiers would avoid double counting the number of patients who have been tested/ treated and improve follow-up with patients referred to other services such as ART or even truly LTFU patients. Until now, and largely due to the success of ART programs, insufficient attention has been given to the establishment of necessary linkages and integration of HIV care within the broader health system; however, our findings indicate that this is an important area of focus as we move towards reaching ‘90-90-90’ and ending the HIV epidemic. Strategies that encourage patients to be more involved in discussions with health care workers about their care may reduce unnecessary transfers due to poor staff attitude which leads to disengagement and transfer to other facilities [7, 24, 29].

### Limitations

In this study, we were limited to only one verification visit of transfers at the receiving facility, hence further follow up with unverified transfers to understand why our verification was unsuccessful would be necessary in order to ascertain if there were any reasons for disengagement. It is possible that the unverified population may not have given us accurate information on the facility they transferred to perhaps due to their experience of being reprimanded for delayed return to care [24]. Additionally, further follow-up in our verified transfer population could reveal important lessons regarding potential long-term impacts associated with clinic transfer- but was not possible in the present study. Although we collected the distance between original and new facility, our study could be further strengthened by a detailed description of the distance from their home. In addition, we could have collected data about patients already on a particular ART regimen being restarted on a different regimen when they present to a new clinic.

Third, our relatively small sample size precluded from further stratification to assess for less prominent associations. Finally, it may be that the unverified patients never transferred to a new facility but reported to the tracer to have done so due to social desirability bias. While our failure to confirm patient transfer at the receiving site could have been due to any one or combination of these reasons, our inability to verify a large proportion (~ 50%) of these patients at all, even after undertaking exhaustive tracing efforts, highlights an important lesson. Our study described transfers among those who accessed care, but the self-reported transfers of those who did not access care is not represented; thus, the transfers within our study population—whether provincial or cross provincial is likely higher than we found through our tracing activities. Lastly, our sample size is not representative of all transfers but only those that were not in EMR at original facility.

## Supporting information

S1 Fig Direct acyclic graph. Direct acyclic graph to identify confounders of ART initiation after presentation at a new site.
(TIF)

## References

[1] Sikazwe I, Eshun-Wilson I, Sikombe K, Czaicki N, Somwe P, Mody A, et al Retention and viral suppression in a cohort of HIV patients on antiretroviral therapy in Zambia: Regionally representative estimates using a multistage-sampling-based approach. PLOS Medicine. Public Library of Science; 2019;16: 1–17. 10.1371/journal.pmed.1002811 31150380 PMC6544202

[2] Clouse K, Vermund SH, Maskew M, Lurie MN, MacLeod W, Malete G, et al Mobility and Clinic Switching Among Postpartum Women Considered Lost to HIV Care in South Africa. JAIDS Journal of Acquired Immune Deficiency Syndromes. 2017;74 Available: http://journals.lww.com/jaids/Fulltext/2017/04010/Mobility_and_Clinic_Switching_Among_Postpartum.6.aspx 10.1097/QAI.0000000000001284 28225717 PMC5324708

[3] Clouse K, Phillips T, Myer L. Understanding data sources to measure patient retention in HIV care in sub-Saharan Africa. International Health. 2017;9: 203–205. 10.1093/inthealth/ihx024 28810667 PMC5881262

[4] Hickey MD, Omollo D, Salmen CR, Mattah B, Blat C, Ouma GB, et al Movement between facilities for HIV care among a mobile population in Kenya: transfer, loss to follow-up, and reengagement. AIDS Care. Taylor & Francis; 2016;28: 1386–1393. 10.1080/09540121.2016.1179253 27145451 PMC5697146

[5] Labonté R, Sanders D, Mathole T, Crush J, Chikanda A, Dambisya Y, et al Health worker migration from South Africa: Causes, consequences and policy responses. Human Resources for Health. 2015; 10.1186/s12960-015-0093-4 26635007 PMC4669613

[6] Camlin CS, Snow RC, Hosegood V. Gendered Patterns of Migration in Rural South Africa. Population, Space and Place. 2014;20: 528–551. 10.1002/psp.1794 25332690 PMC4201383

[7] Geng EH, Bangsberg DR, Musinguzi N, Emenyonu N, Bwana MB, Yiannoutsos CT, et al Understanding Reasons for and Outcomes of Patients Lost to Follow-Up in Antiretroviral Therapy Programs in Africa Through a Sampling-Based Approach. JAIDS Journal of Acquired Immune Deficiency Syndromes. 2010;53: 405–411. 10.1097/QAI.0b013e3181b843f0 19745753 PMC3606953

[8] Geng EH, Odeny TA, Lyamuya R, Nakiwogga-Muwanga A, Diero L, Bwana M, et al Retention in Care and Patient-Reported Reasons for Undocumented Transfer or Stopping Care Among HIV-Infected Patients on Antiretroviral Therapy in Eastern Africa: Application of a Sampling-Based Approach. Clinical Infectious Diseases. Oxford University Press; 2016;62: 935–944. 10.1093/cid/civ1004 26679625 PMC4787603

[9] Fox MP, Bor J, Brennan AT, MacLeod WB, Maskew M, Stevens WS, et al Estimating retention in HIV care accounting for patient transfers: A national laboratory cohort study in South Africa. PLOS Medicine. Public Library of Science; 2018;15: e1002589 Available: 10.1371/journal.pmed.1002589 29889844 PMC5995345

[10] Farahani M, Vable A, Lebelonyane R, Seipone K, Anderson M, Avalos A, et al Outcomes of the Botswana national HIV/AIDS treatment programme from 2002 to 2010: A longitudinal analysis. The Lancet Global Health. Elsevier; 2014;2: e44–e50. 10.1016/S2214-109X(13)70149-9 25104635

[11] Kwong-Leung Yu J, Tok T-S, Tsai J-J, Chang W-S, Dzimadzi RK, Yen P-H, et al What Happens to Patients on Antiretroviral Therapy Who Transfer Out to Another Facility? PLOS ONE. Public Library of Science; 2008;3: e2065 Available: 10.1371/journal.pone.0002065 18446230 PMC2323595

[12] World Health Organization. Guidelines Guideline on When To Start Antiretroviral Therapy and on Pre-Exposure Prophylaxis for Hiv. World Health Organization 2015; 78. 978 92 4 150956 5 26598776

[13] Vincenty T. Direct and Inverse solutions of geodesics on the ellipsoid with application of nested equations. Survey Review. 1975.

[14] Wilkinson LS, Skordis-Worrall J, Ajose O, Ford N. Self-transfer and mortality amongst adults lost to follow-up in ART programmes in low- and middle-income countries: systematic review and meta-analysis. Tropical Medicine & International Health. 2015;20: 365–379. 10.1111/tmi.12434 25418366

[15] Geng EH, Glidden D V, Bwana MB, Musinguzi N, Emenyonu N, Muyindike W, et al Retention in Care and Connection to Care among HIV-Infected Patients on Antiretroviral Therapy in Africa: Estimation via a Sampling-Based Approach. PLOS ONE. Public Library of Science; 2011;6: e21797 Available: 10.1371/journal.pone.0021797 21818265 PMC3144217

[16] Kranzer K, Govindasamy D, Ford N, Johnston V, Lawn SD. Quantifying and addressing losses along the continuum of care for people living with HIV infection in sub-Saharan Africa: a systematic review. Journal of the International AIDS Society. International AIDS Society; 2012;15: 17383 10.7448/IAS.15.2.17383 23199799 PMC3503237

[17] Mugavero MJ, Amico KR, Westfall AO, Crane HM, Zinski A, Willig JH, et al Early Retention in HIV Care and Viral Load Suppression: Implications for a Test and Treat Approach to HIV Prevention. Journal of acquired immune deficiency syndromes (1999). 2012;59: 86–93. 10.1097/QAI.0b013e318236f7d2 21937921 PMC3237801

[18] Luebbert J, Tweya H, Phiri S, Chaweza T, Mwafilaso J, Hosseinipour MC, et al Virological failure and drug resistance in patients on antiretroviral therapy after treatment interruption in Lilongwe, Malawi. Clinical infectious diseases: an official publication of the Infectious Diseases Society of America. 2012;55: 441–8. 10.1093/cid/cis438 22573849

[19] Tweya H, Feldacker C, Estill J, Jahn A, Ng’ambi W, Ben-Smith A, et al Are They Really Lost? “True” Status and Reasons for Treatment Discontinuation among HIV Infected Patients on Antiretroviral Therapy Considered Lost to Follow Up in Urban Malawi. PLOS ONE. Public Library of Science; 2013;8: e75761 Available: 10.1371/journal.pone.0075761 24086627 PMC3784425

[20] Gill MJ, Ody M, Lynch T, Jessiman-Perreault L, Krentz HB. Maintaining the continuity of HIV-care records for patients transferring care between centers: Challenges, workloads, needs and risks. AIDS Care—Psychological and Socio-Medical Aspects of AIDS/HIV. 2016;28: 1073–1078. 10.1080/09540121.2016.1139042 26829326

[21] Stime KJ, Garrett N, Sookrajh Y, Dorward J, Dlamini N, Olowolagba A, et al Clinic flow for STI, HIV, and TB patients in an urban infectious disease clinic offering point-of-care testing services in Durban, South Africa. BMC Health Services Research. 2018; 10.1186/s12913-018-3154-2 29751798 PMC5948731

[22] Kinfu Y, Dal Poz MR, Mercer H, Evans DB. The health worker shortage in Africa: are enough physicians and nurses being trained? Bulletin of the World Health Organization. 2009; 10.2471/blt.08.051599 19377719 PMC2654639

[23] Schneider H, Blaauw D, Gilson L, Chabikuli N, Goudge J. Health Systems and Access to Antiretroviral Drugs for HIV in Southern Africa: Service Delivery and Human Resources Challenges. Reproductive Health Matters. 2006; 10.1016/S0968-8080(06)27232-X 16713875

[24] Mwamba C, Sharma A, Mukamba N, Beres L, Geng E, Holmes CB, et al ‘They care rudely!’: resourcing and relational health system factors that influence retention in care for people living with HIV in Zambia. BMJ Global Health. BMJ Specialist Journals; 2018;3: e001007 10.1136/bmjgh-2018-001007 30483408 PMC6231098

[25] Ware NC, Wyatt MA, Geng EH, Kaaya SF, Agbaji OO, Muyindike WR, et al Toward an Understanding of Disengagement from HIV Treatment and Care in Sub-Saharan Africa: A Qualitative Study. PLOS Medicine. Public Library of Science; 2013;10: e1001369 10.1371/journal.pmed.1001369 23341753 PMC3541407

[26] World Bank. The Role of Digital Identification for Healthcare: The Emerging Use Cases Identification for Development (ID4D) World Bank [Internet]. 2018 Available: www.worldbank.org

[27] Holmes CB, Sikazwe I, Sikombe K, Eshun-Wilson I, Czaicki N, Beres LK, et al Estimated mortality on HIV treatment among active patients and patients lost to follow-up in 4 provinces of Zambia: Findings from a multistage sampling-based survey. Rosen S, editor. PLOS Medicine. Public Library of Science; 2018;15: e1002489 10.1371/journal.pmed.1002489 29329301 PMC5766235

[28] Nglazi MD, Lawn SD, Kaplan R, Kranzer K, Orrell C, Wood R, et al Changes in programmatic outcomes during 7 years of scale-up at a community-based antiretroviral treatment service in South Africa. Journal of acquired immune deficiency syndromes (1999). 2011;56: e1–e8. 10.1097/QAI.0b013e3181ff0bdc 21084996 PMC3776048

[29] Zanolini A, Sikombe K, Sikazwe I, Eshun-Wilson I, Somwe P, Moore CB, et al Understanding preferences for HIV care and treatment in Zambia: Evidence from a discrete choice experiment among patients who have been lost to follow-up. PLOS Medicine. Public Library of Science; 2018;15: e1002636 10.1371/journal.pmed.1002636 30102693 PMC6089406

