# Supplementary figures and images for: Understanding patient transfers across multiple clinics in Zambia among HIV infected adults
Source: PLoS One. 2020 Nov 4;15(11):e0241477. doi: 10.1371/journal.pone.0241477 (PMC7641414; doi:10.1371/journal.pone.0241477)

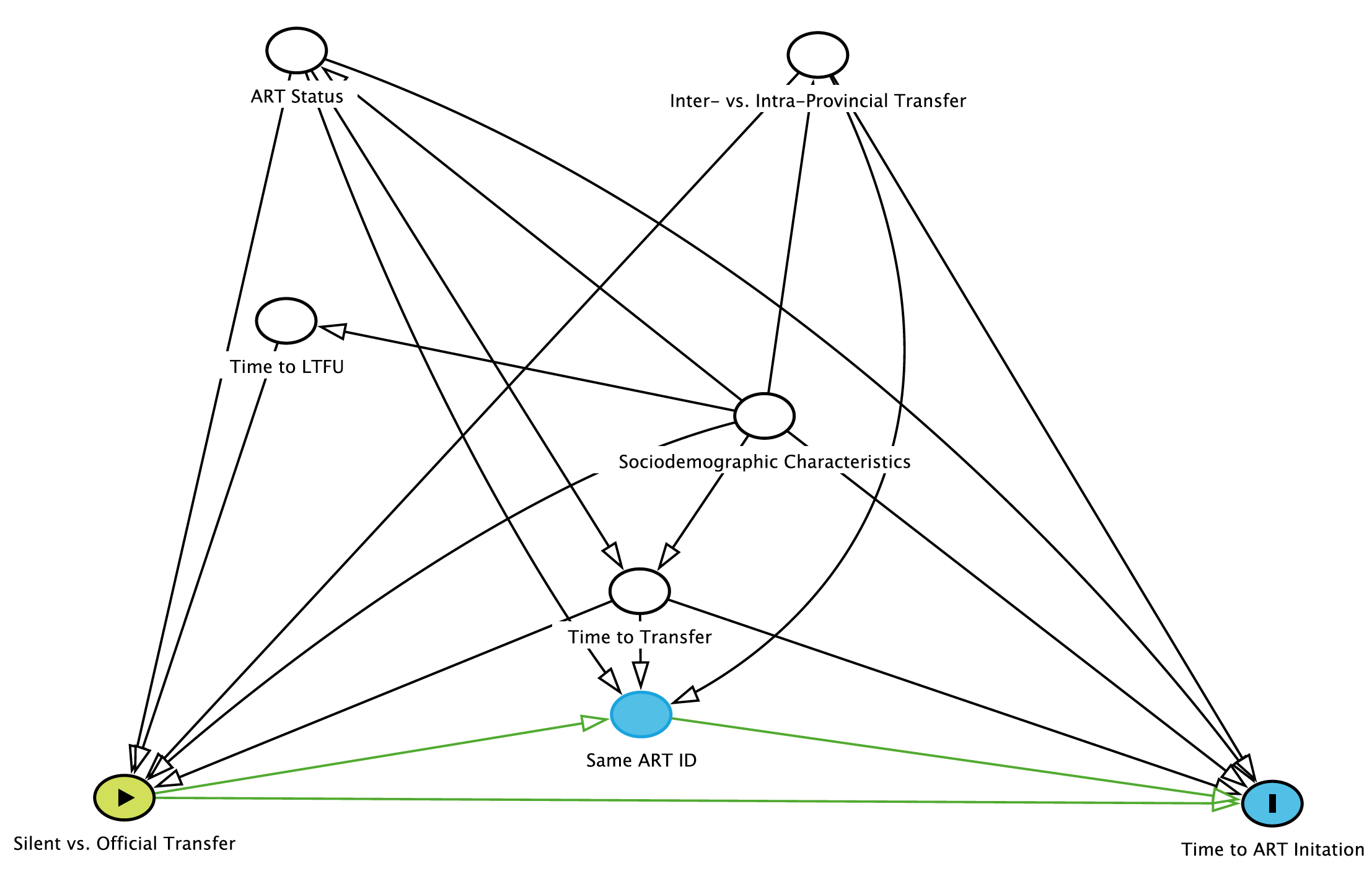

Supplement: S1 Fig — Direct acyclic graph to identify confounders of ART initiation after presentation at a new site. (TIF) [file pone.0241477.s001.tif]
